# Supplementary material for: Examining Physicians’ Approaches to Treating Relatives in Primary Health Care Centers: Insights from a Qualitative Study
Source: Healthcare (Basel). 2024 Oct 11;12(20):2021. doi: 10.3390/healthcare12202021 (PMC11507387; doi:10.3390/healthcare12202021)
Supplement: Supplementary file 1 [file healthcare-12-02021-s001.zip › healthcare-3212095-Topic Guide.pdf]

physicians' perspective about treating family members in primary health care centers: a qualitative study - Focus Group Topic Guide
1 Jan 2023

I want to thank you for taking the time to meet with me today. I would like to talk to you about your perspective and experience regarding treating family members and close friends, the difficulties, and the advantages.

The focus group should take less than 60 min. I will be recording the session because I do not want to miss out on any of your comments. I will be taking some notes during the session. All responses will be kept confidential. This means that your answers will only be shared with research team members, and we will ensure that any information we include in our report does not identify you as the respondent. Remember, you do not have to talk about anything you do not want to, and you may end the interview at any time.

If you can introduce yourself, how many years of experience you have, and mention that you agree to participate in this Focus group.

I would like to start with a scenario
"Imagine you are at a Family gathering and your cousin approaches you and asks to speak to you in private? Your cousin then asked for a favor to facilitate an appointment for him with a Dermatologist"

- Have you ever experienced such a situation? and medical requests from Family members or close friends? Can you share this experience?
- What other kinds of favors/ requests have you been asked or seen other physicians been asked?
- What makes you fulfil / refuse their favor/ request?
- What Advantages and Disadvantages of answering favors/requests?
- Where do you stop in responding to medical care favors/ requests?
- Do you feel treating nonpatients has any influence on the quality of care, compared to treating a regular patient?

Closing Question: "Do you think there is benefit for guidelines/education/training on this topic?"

That is all the questions I have for you; is there something else that we have not covered that you would like to say?

Thank you!
